# Edge Caching Based on Collaborative Filtering for Heterogeneous ICN-IoT Applications

**DOI:** 10.3390/s21165491

**Published:** 2021-08-15

**Authors:** Divya Gupta, Shalli Rani, Syed Hassan Ahmed, Sahil Verma, Muhammad Fazal Ijaz, Jana Shafi

**Affiliations:** 1 Chitkara University Institute of Engineering and Technology, Chitkara University, Rajpura 140401, Punjab, India; divya.gupta@chitkara.edu.in or; 2 Independent Researcher, Corona, CA 13088, USA; sh.ahmed@ieee.org; 3 Department of Computer Science and Engineering, Chandigarh University, Mohali 140413, India; sahilverma@ieee.org; 4 Department of Intelligent Mechatronics Engineering, Sejong University, Seoul 05006, Korea; fazal@sejong.ac.kr; 5 Department of Computer Science, College of Arts and Science, Prince Sattam Bin Abdul University, Wadi Ad-Dwasir 11991, Saudi Arabia

**Keywords:** information centric networking, internet of things, collaborative filtering, edge cloud, content caching

## Abstract

The substantial advancements offered by the edge computing has indicated serious evolutionary improvements for the internet of things (IoT) technology. The rigid design philosophy of the traditional network architecture limits its scope to meet future demands. However, information centric networking (ICN) is envisioned as a promising architecture to bridge the huge gaps and maintain IoT networks, mostly referred as ICN-IoT. The edge-enabled ICN-IoT architecture always demands efficient in-network caching techniques for supporting better user’s quality of experience (QoE). In this paper, we propose an enhanced ICN-IoT content caching strategy by enabling artificial intelligence (AI)-based collaborative filtering within the edge cloud to support heterogeneous IoT architecture. This collaborative filtering-based content caching strategy would intelligently cache content on edge nodes for traffic management at cloud databases. The evaluations has been conducted to check the performance of the proposed strategy over various benchmark strategies, such as LCE, LCD, CL4M, and ProbCache. The analytical results demonstrate the better performance of our proposed strategy with average gain of 15% for cache hit ratio, 12% reduction in content retrieval delay, and 28% reduced average hop count in comparison to best considered LCD. We believe that the proposed strategy will contribute an effective solution to the related studies in this domain.

## 1. Introduction

In recent years, the advancements in the internet of things (IoT) technology has gained a lot of popularity. Today, investigation on different forms for providing IoT as a solution is attracting both industry and academia, as well as seeking attention more than ever [1,2]. Unlike Wireless Sensor networks (WSNs), IoT offers tremendous scope for nodes and their connections. The recent progress ensures easy utilization of IoT in various applications, which include, but are not limited to, smart grids, smart education, intelligent transportation systems, e-healthcare, smart industries, smart agriculture, smart cities, smart homes, and wearables [3]. Due to the plethora of applications support from various disciplines, IoT has now become a bridge between human and real world for information communication. These extensive IoT applications, with almost different nature, have introduced complications in existing wireless communication systems and have, therefore, formed a heterogeneous IoT for today’s real information world. Although the current developments in mobile communication, with its newly launched 5G, offer enhanced mobile broadband (eMBB), massive machine type communication (mMTC), and ultra-reliable and low latency communication (URLLC), still, these heterogeneous IoT always possess higher requirements in terms of reliable connection, low cost, high speed, minimum delay, and scalable communication [4]. Due to limited resource constraints, such as storage and computing with IoT devices, these complex and diverse requirements of heterogeneous IoT tasks can be computed by effective utilization of cloud computing technology, where a network cloud has abundance of these resources. The ever-increasing growth in the IoT technology has offered various advanced functions to several IoT devices. For example, a smart phone today can perform various computations that was earlier possible only using computers or laptops. This simply corresponds to cloud computing in close proximity to users. However, with the massive data being generated by these heterogeneous IoT devices and extremely high latency offered during IoT to cloud communication, the simultaneous access by several IoT devices have demanded high bandwidth requirements. Therefore, the conventional single cloud computing model cannot satisfy all these requirements to meet quality of experience (QoE) [5]. The edge computing has been extended as a layer between cloud and IoT in this process of advancements to provide significant solution [6]. The edge cloud offers sufficient resources for computation and storage requirements [7]. Indeed, the different features offered by this edge computing model has provided various solutions in different domains, but it still faces different challenges, and research in this field is in its infancy [8]. The edge computing itself lacks intelligent computation and communication. The intelligent decision making for different computations based on different scenarios can be implemented by deploying artificial intelligence (AI) technology in the edge cloud and central cloud. The intelligent algorithms were generally deployed on these clouds to make them work smarter. The various fields where AI has proved its effectiveness include robotics, image processing, natural language processing, speech recognition, and so on [9]. Recently, cloud computing has started using AI’s cognitive services to offer better QoE. Moreover, the delays involved in current internet architecture for IoT content distribution due to its IP address-based approach where content has to be fetched from target device offers various challenges. This can be managed efficiently by leveraging information centric networking (ICN) communication in IoT and is referred as ICN-IoT [10,11].

To facilitate enhanced network performance, this study aims to achieve faster content distribution for IoT device user’s requested content in support to higher cache hit ratio, reduced delay, and low path stretch. The integration of ICN with IoT would support fast searching based on content names, as well as caching of content, within network nodes. The edge cloud would offer low overhead to central cloud for request processing, as well as low delay to user requests. The AI deployed in edge cloud would be utilized for making intelligent decision in regards to requested content caching on edge nodes. Our proposed solution for effectively managing the massive traffic flows on the central cloud mainly considers caching of frequently requested contents at edge clouds which are in near proximity to IoT end users. The proposed solution leverages collaborative filtering and k-means clustering techniques for making intelligent caching decisions at edge nodes. Indeed, various studies in literature do exist for dealing with these challenges. Similar to this, the authors in References [12,13] proposed use of small distributed data centers in entire network to reduce burden at core cloud network, and some works [14,15,16] offer prefetching of content at edge nodes to alleviate and control back-haul traffic. Although the concept of collaborative filtering was utilized by various online business applications, websites and live streaming services for generating recommendations for user’s preferences, yet, found its application in networking domain after the proposal presented in Reference [17]. The author presented effective benefit for content placement decision based on collaborative filtering. However, this could also be used as a improved solution for various other challenges, such as traffic bottlenecks, bandwidth wastage, and timely content delivery. Despite its several uses in various applications [18,19,20], item-based collaborative filtering in support to better QoE is still not properly utilized in networking and communication. In addition, this concept has been mainly implemented in the networks supporting TCP/IP architecture, which further degrades system performance due to its target-based delivery approach.

Considering benefits associated with the combination of all these technologies, such as ICN-IoT, cloud computing, and edge computing, along with the deployment of AI, our main focus is on designing AI-enabled edge model for intelligent content caching strategy which is suitable for heterogeneous IoT architectures to effectively manage massive traffic flow on central clouds.

To this end, following are the contributions made in this paper:

- We propose an enhanced ICN-IoT content caching strategy by enabling AI’s collaborative filtering within edge cloud to support heterogeneous IoT architectures for traffic management at conventional cloud computing model. An architecture is designed by combining ICN-IoT, edge, cloud, and AI for heterogeneous IoT applications to provide an enhanced hardware model which support user’s QoE.
- We propose a content-based collaborative filtering caching technique for intelligently caching content on edge nodes. Through the combination of a wireless communication model, and collaborative filtering caching model of edge nodes, a content fetching algorithm is designed to retrieve user’s data efficiently.
- We perform extensive simulations to validate the effectiveness of our proposed scheme over state-of-the-art caching strategies, such as LCE, LCD, ProbCache, and CL4M. The results obtained from experimentation prove that our proposed scheme significantly achieves higher cache hit ratio. In addition, the proposed scheme is efficient to achieve lower content delay and reduced path stretch when compared to these strategies.

The rest of this paper is structured as follows: Section 2 provides brief discussion on the related work in this domain. The system model of our proposed strategy, along with its major components, is presented in Section 3. In Section 4, the detailed description of our proposed design, while considering content caching based on collaborative filtering, is given. The performance of the proposed approach is evaluated in Section 5. Finally, the conclusion of this study is presented in Section 6.

## 2. Related Work

This section represents some recent studies in relation to IoT, ICN, edge computing, and artificial intelligence, either as an individual technology or in combination for designing efficient caching strategies. The studies of these works would be beneficial to justify our motivation for conducting this study and support academic achievements in the research field.

### 2.1. IoT and ICN

With ever-increasing traffic on the internet due to several connected IoT devices, the management of each IP-based content request following conventional network architecture has started to impose a challenge for its performance. Combining IoT with ICN, the work in Reference [21] focused on incorporating ICN in IoT. The benefits gained by IoT from ICN and various challenges being addressed through this combined architecture is introduced in this study. The works in Reference [21] continued research in this combined field and addressed various issues in this combined architecture by introducing ICN-based IoT architecture. Further, work in Reference [22] listed comparison of IoT integration with different CCN standards and offered some improvements towards data traffic management. In order to reduce the energy consumption in IoT networks, an ICN-based forwarding strategy for data transmission was proposed by Reference [23]. The work in Reference [24] investigated a unique naming scheme for smooth interest packet flow between NDN-IoT-based regions. Similarly, management of the data packet overhead was discussed in Reference [25]. Similarly, working towards energy efficiency, the work in Reference [26] proposed an ICN by combining with specific IoT approach called TSCH. Further, the authors in Reference [27] proposed a context-based approach for IoT data communication utilizing ICN architecture. The approach mainly focused on correct routing and forwarding of information with management of FIB and PIT data structures. Moreover, Quevedo et al., in Reference [28], presented various caching strategies while considering bandwidth and energy issues faced by this integration. Generally, cache schemes focus on providing solution to three issues namely which location is best suited to cache data, what content should be cached and how the content to be stored. The default caching scheme is Cache Everything Everywhere (CEE) [29], where each intermediate node locally caches each piece of content which passes through it. The policy is straightforward but results in high content redundancy. The simple location-based caching is Leave Copy Down (LCD) [30], which locates content at the node, i.e., one level below where cache hit occurs along the delivery path. However, due to frequent requests of popular contents, at some instance, all nodes will have a copy of same content, leading to cached content duplication, as well as cache full. The prob(p) [30] is another simple yet stateless caching scheme where content on routers are cached based on some probability. The scheme does not consider the router’s location while making a caching decision. To resolve the location issue, ProbCache provides high probability for caching when content is near to the consumer [31]. The scheme manages to reduce redundancy but at the cost of low cache utilization of the node far from the consumer. To support chunk level caching, WAVE, based on content popularity and nodes co-ordination, was proposed [32]. The approach is similar to LCD and differs by caching content exponentially at the neighbor based on frequency of content. The scheme stores frequent requests near the edge router but does not consider data packet caching time.

The various broad caching strategies, such as probability-based [30,31], popularity aware [33,34], reactive caching [30,32], proactive caching [35,36], and non-cooperative [29,36], were designed for various areas, such as mobile devices, IoT, 5G, vehicular networking, ad hoc networks, etc. However, these strategies do not fit for an environment with limited resources due to their own limitations, in one way or another. For non-cooperative caching, every node individually makes a caching decision on whether to cache content or not, hence resulting in cache redundancy and no effective utilization of resources.

To address the problems in non-cooperative caching, researchers focused on design of cooperative caching schemes for ICN. In cooperative caching, network nodes work in collaboration with others for making caching decision.

### 2.2. IoT and Edge Computing

The support for IoT applications using edge computing model was presented by authors in Reference [37]. The performance comparison in terms of energy efficiency and content fetching delay was conducted to highlight importance of edge in IoT. Further, researchers in Reference [38] focused on challenges faced by current approaches of IoT and recommended use of fog computing in IoT scenarios based on several reasons. The study in Reference [39] analyzed the security aspects offered by IoT-fog computing when compared with IoT-clouds. Sarkar et al., in Reference [40], investigated the suitability of fog computing for meeting the requirements from various heterogeneous IoT applications which, in reality, are not feasible to accomplish with use of traditional cloud model. The work in Reference [41] presented different new approaches for combining in IoT architecture and discussed the benefit of incorporating mobile edge computing (MEC) in IoT, which itself adopted the fog computing model. In addition, the researchers in Reference [42] proposed a model for agreement of resources. The work was mainly focused around achieving efficient resource management and demonstrating its effectiveness after evaluation on the cloudSIM toolkit. The study in Reference [43] proposed a model for supporting reasonable and effective communication among IoT devices. The algorithm used the concept of matching theory for accomplishment of node pairs. Further, the feasibility while combining fog computing with smart gateways was analyzed in Reference [44]. The authors in Reference [45] proposed home-box networks for efficient content delivery in peer to peer overlay networks. The design architecture considered several delays to improve the service performance in the IoT domain.

Moreover, given limitations offered due to inflexible design of Fog-IoT architecture to meet current demands, the work in Reference [21] proposed smart collaborative caching by leveraging ICN for IoT in a fog environment. the solution was designed to achieve content caching, node location tracing, and resource sharing. Further, the authors in Reference [46] proposed a joint optimization solution for fog-IoT networks which basically deals with issues related to content caching, computation offloading, and resource sharing. The paper proposed a solution based on actor-critic-reinforcement learning to solve joint optimization issues. Similarly, working on 3C, i.e., computation, caching, and communication, Luo et al. [47] proposed an efficient algorithm based on an iterative task team formulation method for solving these issues as a subproblem, with minimum possible cost. The researchers in Reference [11] proposed a fog-based caching scheme in an IoT environment by utilizing ICN. The proposed solution worked towards offering minimum latency to user content requests by providing content near to edge networks based on its popularity. Working in the same direction to offer minimum service delay to IoT nodes while reducing energy consumption, work in Reference [48] proposed smart clustering mechanism by utilizing both fog nodes (FNs) and terminal nodes (TNs).

### 2.3. Artificial Intelligence in Edge Caching

The artificial intelligence in terms of machine learning and deep learning can be applied to wireless edge networks for deciding what content to cache and where to cache so that caching objectives are optimized. The authors in Reference [49] proposed a caching scheme which predicts the popularity of the new video based on the similarity of features which are already present in the published video. The work in Reference [50] proposed popularity-based supervised and deep learning framework for caching at base stations in mobile edge computing networks. Further, the work in Reference [51] considered learn-to-rank algorithm and k-means clustering for caching content in small networks. The scheme aims to maximize cache hit ratio (CHR), and the solution of optimization problem is NP-hard if content popularity is not known. The algorithm was designed based on historical content requests. The works in References [52,53] presented proactive learning-based caching at small base stations and user equipment to meet user satisfaction ratio. Chang et al. [54] presented a big data and ML-based framework for caching content inside edge networks. The smart caching in edge networks was explained using two case studies, where the first was designed by combining unsupervised learning and deep learning, and the second was using social ties between end users. The reinforcement learning is also used by many studies for deciding content caching. The learning process of an RL agent can be modeled as optimal control of a Markov Decision Process (MDP). Based on this, the work in Reference [55] presented base station-based distributed caching and delivery framework. The cache replacement transmission was minimized using an MDP optimization solution based on variables, such as popularity and transmission cost of cache replacement from one base station to another. The model uses the Q-learning approach for transmission cost minimization. The authors in Reference [56] proposed deep reinforcement learning approach for content caching and network slicing in vehicular environment. The work in Reference [57] proposed multi-tier content caching based on deep Q-learning to support improved performance in radio access networks. The comparison of different works based on several parameters is represented in Table 1.

## 3. System Model

**Figure 1:**
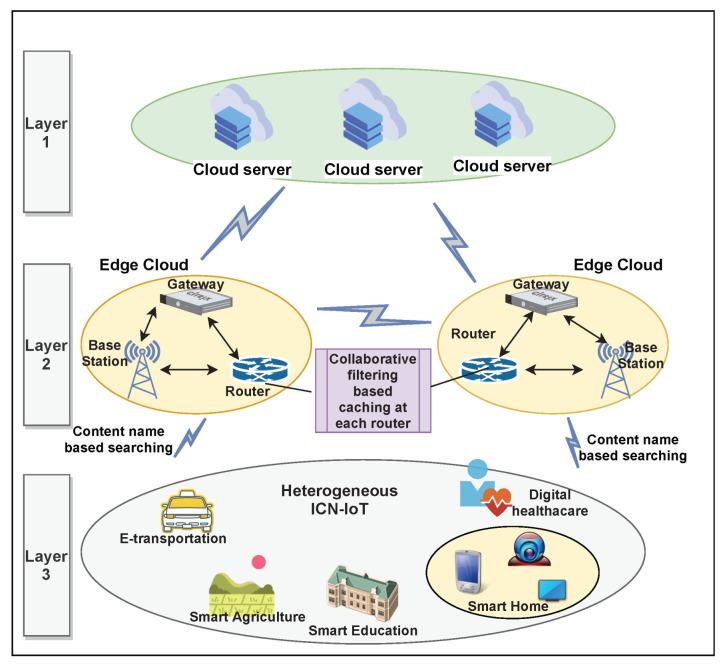
Three-tier network architecture.

This paper considers an ICN-based heterogeneous IoT environment. The users in this network can send message to content providers to receive required content [4]. A three-tier network architecture serving various IoT applications is presented in Figure 1. Layer 1, also known as the top layer, represents the core network comprising various cloud servers. These servers are assumed to have all the data demanded by the users. In addition, these servers maintain records of all access history from various edge nodes (ENs) that it serves. The middle layer (layer 2) is mainly comprised of several ENs, where each EN is connected with number of end users. The several users from diversified ICN-IoT applications constitute the bottom layer of this architecture. Here, we represent all ENs as set $EN=\{EN_{1},EN_{2},\ldots ,EN_{M}\}$ for *M* number of total available ENs in the network. The users in the network are represented as set $U=\{u_{1},u_{2},\ldots ,u_{K}\}$ for *K* number of total users, where each user may have varying preferences. The cloud server stores total *N* contents and can be represented as set $C=\{c_{1},c_{2},\ldots ,c_{N}\}$, where each piece of content is assumed to have equal size of *T* Mbits. Moreover, each piece of content is supposed to have set *F* of *J* attributes, $F=\{f_{1},f_{2},\ldots ,f_{J}\}$, where each attribute denotes some feature. For example, if content represents clothes, the attribute may specify the genre of the clothes, such as western, fashion, ethnic, indo-western, etc., with each piece of clothing having an index value between 0 and 1 for each genre. For instance, a western piece of clothing with lots of cuts and short length may be assigned larger index value for its style and fashion attributes.

Based on Table 2 and Table 3, for each EN and content, the sum of all the values of the attributes is equal to 1, i.e., $\sum_{i=1}^{J}f_{i}=1$ for every *C* and EN.

To calculate the number of times a specific content $C_{q}$ has been requested by any $EN_{p}$, a history of requests is created, as shown in Table 4. The value of particular cell $REQ_{p,q}$ is, therefore, calculated as:

(1) $$REQ_{p,q}=\frac{EN_{p}*C_{q}}{J}\leq 1.$$

Here, $EN_{p}$ represents rows in Table 2, and $C_{q}$ represents columns in Table 3. On the other hand, the value for cell $REQ_{p,q}$ can also be retrieved from history by setting $REQ_{p,q}$ to the fraction of times content $C_{q}$ has been requested by $EN_{p}$ among all content requests. This REQ is important and would be useful for deciding the content placement inside ENs.

The cloud center is a huge database with capacity equal to vol. All the content requested by IoT user can be provided through a traditional cloud data center but with longer delays due to congestion bottleneck. The edge nodes on edge clouds can cache some content frequently requested by users to offer reduced latency. To provide simplicity to our design approach, we consider homogeneous cache capacity for each edge node. Therefore, we assume each EN is equipped with cache of equal size, say $Size_{L}$, such that

(2) $$Size_{L}=vol*\rho ,$$

where ρ contains a value between 0 and 1, i.e., 0<ρ<1, and vol represents the total capacity of the cloud server database. $Size_{L}$ denotes a very small cache space available to each EN as compared to total volume vol.

In our design model, we consider dividing the cache size of each EN into two equal halves, where one half, i.e., $Size_{L1}=\frac{Size_{L}}{2}$, is used for caching the content based on $EN^{’}s$ local popularity Lp for each EN. $Size_{L1}$ contains a set of Z most popular contents $C_{1},C_{2},\ldots ,C_{Z}$ arranged in descending order of their popularities. The content ranking depends on the value of Pop, where $Pop_{EN_{p},C_{q}}$ represents the popularity of $C_{q}$ in edge node $EN_{p}$. This Pop value can be obtained from REQ (history of requests). The other half of cache space, i.e., $Size_{L2}=Size_{L}-Size_{L1}$, is used to cache V contents based on content-based collaborative filtering technique. Both cache spaces are utilized to perform content placement during off-peak hours so as to minimize network traffic in peak hours.

## 4. Proposed Framework

The AI-enabled content placement and caching among edge nodes to support heterogeneous IoT applications is proposed using collaborative filtering technique.

### 4.1. Edge Clustering

In order to obtain benefit from intelligent content caching strategy, all the edge nodes are partitioned into several clusters [48]. Based on the entries present in REQ (Table 4), where values can be obtained using Equation (1), we apply k-means clustering to group the ENs into *G* clusters (CLs), i.e., $CL_{1},CL_{2},\ldots ,CL_{G}$ [58]. We use cosine distance metric (refer to Equation (3)) to calculate the distance between any pair of ENs, say $EN_{i}$ and $EN_{j}$[52]. ENs which belong to the same cluster are going to have distance values approximately near to 0, whereas ENs belonging to different clusters will have distance value near to 1. Moreover, the zero value represents the EN is locating exactly in the middle of two CLs.

(3) $$dist\left(EN_{i},EN_{j}\right)=1-\frac{\sum_{m=1}^{N}REQ_{i,m}REQ_{j,m}}{\sqrt{\sum_{m=1}^{N}{\left(REQ_{i,m}\right)}^{2}}\sqrt{\sum_{m=1}^{N}{\left(REQ_{j,m}\right)}^{2}}}.$$

Here, $REQ_{i,m}$ represents the number of requests $EN_{i}$ has made for content $C_{m}$.

Further, to decide the optimal number of CLs, there are various methods already available in the literature. We used Silhouette coefficient method as a metric for deciding the efficiency of clustering method [59,60]. This method is generally used to estimate how much certain observation fits to its cluster. To decide the optimal number of clusters, the average silhouette coefficient is calculated for every possible number of clusters, and the one with the highest average silhouette coefficient value is chosen.

### 4.2. Edge Caching

As discussed in the previous section, each ED is equipped with certain cache space, i.e., $Size_{L}$. Out of total available space, one portion, i.e., $Size_{L}1$, is used to cache contents based on the local popularity of the content. However, the rest of the portion $Size_{L}2$ is used to cache content based on the highest probability of content to be requested in future. To predict the probability of future requests for available contents, we apply content-based collaborative filtering. To proceed in this direction, firstly, the similarity index between any two pair of contents says $C_{i}$ and $C_{j}$ is being calculated using cosine coefficient as given in Equation (4) [53].

(4) $$Sim\left(C_{i},C_{j}\right)=\frac{C_{i}*C_{j}}{\|C_{i}\left\|*\right\|C_{j}\|}=\frac{\sum_{b=1}^{J}C_{b,i}*C_{b,j}}{\sqrt{\sum_{b=1}^{J}{\left(C_{b,i}\right)}^{2}}\sqrt{\sum_{b=1}^{J}{\left(C_{b,j}\right)}^{2}}}.$$

Here, $C_{i}$ and $C_{j}$ are the *i*th and *j*th contents being represented as a column in Table 3, and *b* is the *b*th feature from total available *J* number of features. In contrast to the cosine distance coefficient, cosine similarity coefficient has value 1 when two contents are similar. The value 0 represents no similarity between two contents. The similarity index between the same file will always result to value 0, i.e., $sim(C_{i},C_{i})=0$ for all *i* (refer to Table 5).

Using Table 4 and Table 5, we construct a content prediction table CP (Table 6), where $CP_{i,j}$ can be calculated using formula:

(5) $$CP_{i,j}=\sum_{d=1,d\neq j}^{N^{\prime }}sim\left(C_{d},C_{j}\right)*REQ_{i,d}.$$

Here, $N^{\prime }$ represents set of all the contents requested by $ED_{i}$. The higher value of $CP_{i,j}$ represents high probability of content $C_{j}$ being requested by edge node $ED_{i}$. Based on the prediction results, $ED_{i}$ will cache contents with high future request probability in to its $Size_{L2}$ space.

### 4.3. Content Fetching

Based on the content caching scheme discussed above, each ED caches content in its allocated space. Each user $U_{x}$ must be associated with at least one ED, based on the nearest distance rule. Considering a request for a content $C_{i}$ by user $U_{x}$ from an edge device $ED_{j}$, the local cache of $ED_{j}$ would be checked first to determine if it contains requested content $C_{i}$. In case of a match, $C_{i}$ would be delivered to $U_{x}$ locally. On the other hand, if $ED_{j}$ does not cache a copy of $C_{i}$, it will start searching $C_{i}$ among all the EDs belonging to its own cluster $CL_{s}$. If $C_{i}$ is cached by any of the EDs belonging to $CL_{s}$, it would be delivered to $U_{x}$ without caching in to $ED_{j}$. Otherwise, in case of search failure inside $CL_{s}$, the content would be requested from central cloud server and would be cached locally inside $ED_{j}$, based on LRU replacement policy. Algorithm 1 explains the content fetching procedure. The likelihood of the content requests generated by users is based on the history of requests (refer to Table 4). Each user would be connected to same ED for a duration that is long enough to allow full content delivery.
**Algorithm 1:** Content Searching $C_{i}$.**Input**: $U_{x}$ associated with $ED_{j}$**Output**: $C_{i}$**Begin:**  1   For each $C_{i}$ requested by $U_{x}$ do  2   Check $C_{i}$ in $ED_{j}$’s cache  3   if ((($Size_{L1},Size_{L2}$) ← check ($C_{i}$) ) ≠ 1)  4        if(($CL_{s}$ ← check ($C_{i}$)) ≠ 1)  5           cloud Database ← check($C_{i}$)  6              $ED_{j}$ caches $C_{i}$  7               return $C_{i}$ to $U_{x}$  8        else  9           return $C_{i}$ to $U_{x}$  10        endif  11   else  12        return $C_{i}$ to $U_{x}$  13   endif**End**

## 5. Evaluation Scenario

The efficiency of the proposed strategy has been evaluated against ICN-IoT benchmark caching schemes by implementing simulations in Icarus simulator. The simulator with four building blocks, such as scenario generation, scenario orchestration, experiments execution, and result collection, is specifically designed for research in field of ICN caching and routing. Before evaluation, network is initially warm up with $3\times 10^{5}$ number of requests. The warm-up requests are initial messages which are sent to network caching nodes to perform content caching before analyzing system performance. To measure performance evaluation, measured requests are set to $6\times 10^{5}$ and are sent in network after completion of warm-up phase. Each user sends request messages that follow Poisson distribution as this is the most widely used distribution by various caching strategies during implementation. The network request rate is set to default value as in Icarus settings, i.e., 12 requests per second for the whole network. The content popularity follows Zipfian distribution, with skewness parameter α ranges from 0.6 to 1.2. In Zipfian distribution, the value α corresponds to concentration of user preference. The large α value signifies higher concentration of user preferences; in case of α = 1.25, more users are interested in same request in contrast to α = 0.8. The experiments are performed using tree network topology as this is highly preferred for performance evaluation in recent works. To maintain the fairness, the uniform storage capacity is allocated to each node by dividing total network caching capacity with the number of contents. For experimentation purpose, this study considers different scenarios by varying network cache from 0.04% to 5%. In addition to content placement scenario generation, the replacement of content is also important. The proposed strategy considers LRU for cache replacement due to its low complexity and high consistency with already available ubiquitous caching schemes. Based on all the aforementioned settings, the experiments were performed for 4 different content popularity (α) values with consideration of 4 network cache capacities for each network topology. The simulations was performed twice, and the average value for each performance metric was considered to compare the proposed strategy with other benchmark schemes.

### 5.1. Performance Metrics

From the wide variety of metrics available for computing significance of caching strategies [61], this work evaluates the performance of proposed strategy based on the following:

#### 5.1.1. Cache Hit Ratio (CHR)

CHR determines the ratio of number of requests processed by edge caching nodes rather than cloud servers. Assuming total M requests being served by network, if request for any content k is cached inside an edge node and can be served to requester, then it is counted as cache hits ($Cache_{hits}$). In case of requested content not found in edge node, it is served by a cloud server and is considered as cache miss ($Cache_{miss}$) The formula for calculating CHR is:

(6) $$Cache\;Hit\;Ratio\;\left(CHR\right)=\frac{Cache_{hits}}{Cache_{hits}+Cache_{miss}}.$$

#### 5.1.2. Content Retrieval Delay (CRD)

CRD refers to the total delay incorporated in getting the requested content k by an end user. In order to calculate total delay, the forwarding operation of both request message for content k and response message with content object K are considered. Therefore, CRD is computed using the formula given in the following equation.

(7) ContentRetrievalDelay(CRD)=requesttraveldelay+responsetraveldelay.

#### 5.1.3. Average Hop Count (*AHC*)

*AHC* defines the average number of hops a content request needs to travel in order to be satisfied when normalized over total hops until reaching original server. The 0 value of AHC tends to requests that are served more closely to user, hence the caching strategy shows its highest efficiency. The value of AHC can be computed using the formula given in the following equation.

(8) $$Average\;Hop\;Count\;\left(AHC\right)\;=\;\frac{Number\;of\;hops\;travelled}{Number\;of\;hops\;to\;server}.$$

### 5.2. Simulation Results

The evaluation results of the proposed strategy has been compared against various benchmark caching schemes, which include LCE, LCD, CL4M, and ProbCache. This section represents the results obtained after performing simulations on aforementioned caching strategies for different performance metrics.

#### 5.2.1. Cache Hit Ratio (CHR)

**Figure 2:**
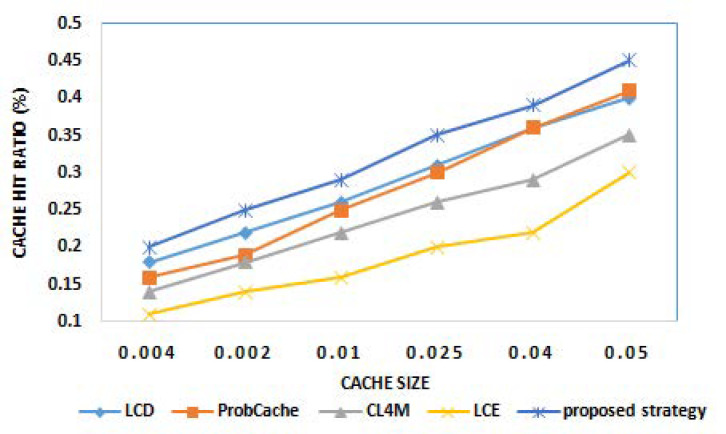
Cache hit ratio for different cache sizes.

**Figure 3:**
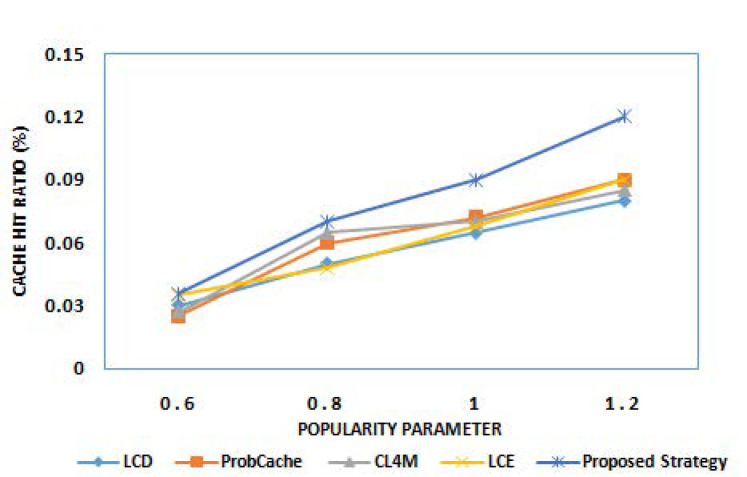
Cache hit ratio for different popularity parameter α.

The cache hit ratio is the most important metric to examine the performance of any caching strategy. It determines the percentage of requests being served out of total generated requests. The high CHR always implies reduced burden on the core network as most of the requests are served by intermediate nodes. The initial round of experiments examine the performance of the proposed strategy for CHR. Figure 2 and Figure 3 show the cache hit ratio results of various caching strategies for different cache size and different popularity parameter α, respectively. From the results obtained, it can be observed that the proposed strategy outperforms existing benchmark caching strategies for higher cache hit operations. The reason behind the better performance of the proposed strategy, among others, is due to caching content on edge devices based on content popularity and future prediction. With the increasing popularity of requested content, the more content would be cached at edge nodes, and, hence, higher CHR can be attained.

#### 5.2.2. Content Retrieval Delay (CRD)

**Figure 4:**
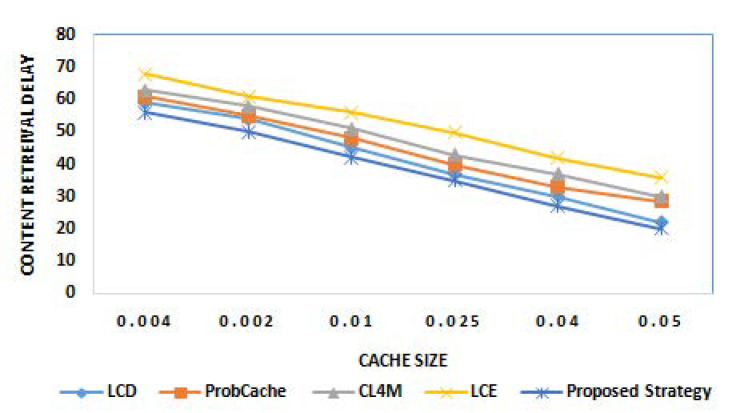
Content retrieval delay for different cache sizes.

**Figure 5:**
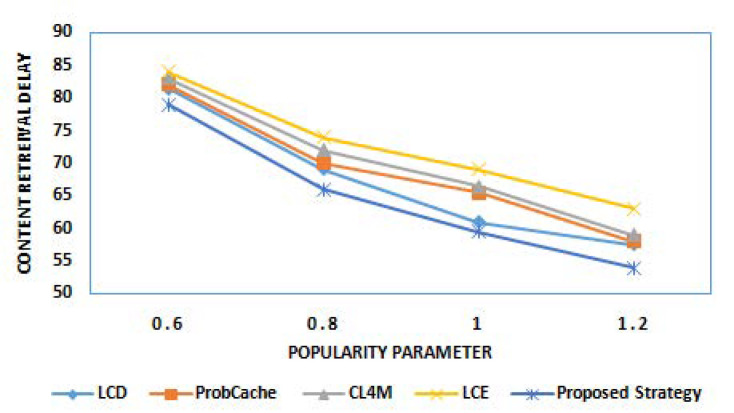
Content retrieval delay for different popularity parameter α.

The next step of experiments work for interpreting the results of content retrieval delay offered by proposed strategy in comparison to benchmark caching strategies. This metric is mainly used to compute network latency. Figure 4 and Figure 5 show the result of content retrieval delay of various caching strategies for different cache size and different popularity parameter α, respectively. The results clearly reveal the outstanding performance of the proposed strategy among other considered strategies for reduced retrieval delay with increase in cache size, as well as content popularity. The reduction in retrieval delay with gradually increasing cache size is due to higher cache capacity of edge nodes to cache content and provides data packets for user requests. Similar to this, less delay is observed for content having higher popularity because of its caching near the user.

#### 5.2.3. Average Hop Count (*AHC*)

**Figure 6:**
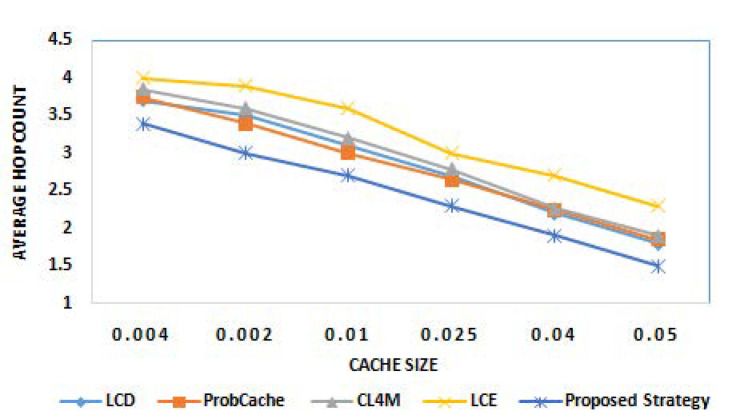
Average hop count for different cache sizes.

**Figure 7:**
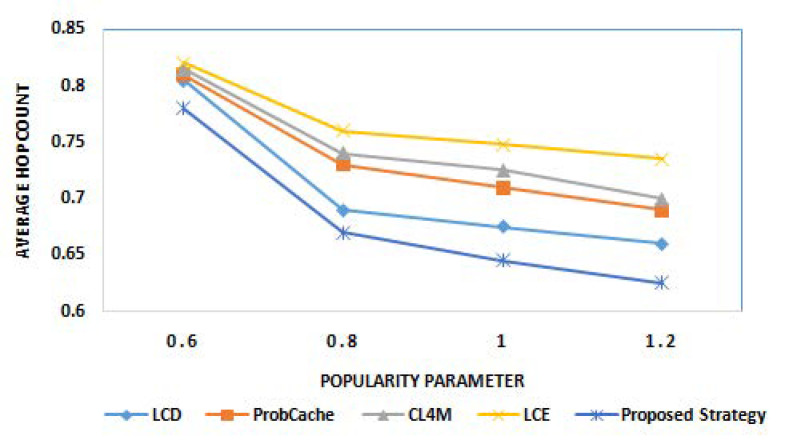
Average hop count for different popularity parameter α.

The last round of experiments investigated the performance of proposed strategy among aforementioned caching strategies, for comparison by considering another challenging metric, i.e., average hop count. Figure 6 and Figure 7 show the average hop count results of various caching strategies for different cache sizes and different popularity parameter α, respectively. From the results obtained, it can be observed that the proposed strategy outperforms existing state of art caching strategies by reducing average number of hops traversed during content delivery operations. For the average hop count, the maximum improvement is recorded in case of proposed strategy (with varying cache size). This is due to the design model of the proposed strategy where caching at intermediate nodes is always aimed to reduce hop count with minimum delay. From the results in Figure 6, the continuous fall in average hop count value with growing cache size can be clearly observed for all caching strategies. This represents the direct significance of cache size on number of hops traversed. The decrease in count of hops with increase in popularity for all strategies can be seen in Figure 7. The caching of the most popular and future predicted content near the user is the reason behind the obtained results.

## 6. Conclusions

In this paper, we aimed at providing better QoE to user by offering minimum content retrieval delay, as well as reduced average number of hops utilized to obtain desired content and to enhance cache hit ratio on each edge node. To this end, we first considered an edge-enabled heterogeneous ICN-IoT network architecture to satisfy user’s latest demands. Further, to support an intelligent caching, we proposed an collaborative filtering-based content caching strategy on each edge cloud where contents would be cached based on its local popularity and predicted future demands. Afterwards, utilizing both edge clustering and caching, an algorithm was designed for fetching content by user from the network to meet QoE. Numerical results revealed the effectiveness of our proposed strategy over proposed strategy over various benchmark strategies, such as LCE, LCD, CL4M, and ProbCache, for achieving considered cache hit ratio and content retrieval delay. The reason behind the improved performance of our strategy in comparison to existing considered benchmark strategies is caching content based on some request history, thereby predicting future demands of users. As the work is carried out in collaborative filtering with various cache sizes, the proposed technique still needs to be checked with various NDN schemes. For the future work, we will design a caching strategy while considering large set of attributes available with the requested content and varying caching capacity of all the edge nodes to further support scalable network with reduced content retrieval latency.

## Figures and Tables

**Table 1:** AI-based caching techniques.

Ref	Machine Learning Technique	Algorithm	Objective	Caching Strategy	Caching Location	Network
[49]	Supervised	CNN	High computation offloading ratio	Proactive	Base station	wireless cellular
[50]	Supervised	DNN	Reduced content retrieval delay	Proactive	Base station	Mobile edge computing
[51]	Supervised and unsupervised	Learn-to-rank	Improved cache hit ratio	Reactive	Small base station	Small cell network
[52,53]	Supervised	NA	High user satisfaction ratio	Proactive	Base station, user equipment	Small cell network
[54]	Unsupervised	DNN	Minimize latency, High data rate	Proactive	Mobile base station	Hetnet
[55]	Reinforcement learning	Q-learning	minimum cache replacement transmission cost	Proactive	Base station, user equipment	Macro cellular
[56]	Reinforcement learning	Deep RL	maximum cache hit ratio	Reactive	V2I	Radio access networks
[57]	Reinforcement learning	Deep Q-learning	maximum cache hit ratio	Proactive	Base station, user equipment, access point	Radio access networks

**Table 2:** EN features.

Edge Nodes/Features	*f* ${}_{1}$	*f* ${}_{2}$	⋯	*f* ${}_{J}$
*EN* ${}_{1}$	⋯	⋯	⋯	⋯
*EN* ${}_{2}$	⋯	⋯	⋯	⋯
⋯	⋯	⋯	⋯	⋯
*EN* ${}_{M}$	⋯	⋯	⋯	⋯

**Table 3:** Content features.

Features/Contents	*C* ${}_{1}$	*C* ${}_{2}$	⋯	*C* ${}_{N}$
f${}_{1}$	⋯	⋯	⋯	⋯
f${}_{2}$	⋯	⋯	⋯	⋯
⋯	⋯	⋯	⋯	⋯
f${}_{J}$	⋯	⋯	⋯	⋯

**Table 4:** REQ: History of requests.

Edge Nodes/Contents	*C* ${}_{1}$	*C* ${}_{2}$	⋯	*C* ${}_{N}$
ED${}_{1}$	⋯	⋯	⋯	⋯
ED${}_{2}$	⋯	⋯	⋯	⋯
⋯	⋯	⋯	⋯	⋯
ED${}_{M}$	⋯	⋯	⋯	⋯

**Table 5:** Content similarity.

Similarity	*C* ${}_{1}$	*C* ${}_{2}$	⋯	*C* ${}_{i}$	⋯	*C* ${}_{j}$	⋯	*C* ${}_{N}$
C${}_{1}$	0	⋯	⋯	⋯	⋯	⋯	⋯	⋯
C${}_{2}$	⋯	0	⋯	⋯	⋯	⋯	⋯	⋯
⋯	⋯	⋯	0	⋯	⋯	⋯	⋯	⋯
C${}_{i}$	⋯	⋯	⋯	0	⋯	⋯	⋯	⋯
⋯	⋯	⋯	⋯	⋯	0	⋯	⋯	⋯

**Table 6:** CP: Content prediction.

Prediction	*C* ${}_{1}$	*C* ${}_{2}$	⋯	*C* ${}_{i}$	⋯	*C* ${}_{j}$	⋯	*C* ${}_{N}$
ED${}_{1}$	⋯	⋯	⋯	⋯	⋯	⋯	⋯	⋯
ED${}_{2}$	⋯	⋯	⋯	⋯	⋯	⋯	⋯	⋯
⋯	⋯	⋯	⋯	⋯	⋯	⋯	⋯	⋯
ED${}_{M}$	⋯	⋯	⋯	⋯	⋯	⋯	⋯	⋯
